# A dataset for the application of the Bayesian AMMI (B-AMMI) model to identify high-yielding and stable barley genotypes

**DOI:** 10.1016/j.dib.2026.113309

**Published:** 2026-10-03

**Authors:** Salim Rahmati, Alireza Pour-Aboughadareh, Reza Azizi-Nezhad, Lia Shooshtari, Alireza Etminan, Bita Jamshidi, Omid Jadidi, Jan Bocianowski

**Affiliations:** a Department of Plant Breeding and Biotechnology, Science and Research Branch, Islamic Azad University, Tehran, 14778-93855, Iran; b Seed and Plant Improvement Institute, Agricultural Research, Education and Extension Organization (AREEO), Karaj, P.O. Box 31587-77871, Iran; c Department of Plant Breeding and Biotechnology, Ker. C., Islamic Azad University, Kermanshah, Iran; d Department of Food Security and Public Health, Khabat Technical Institute, Erbil Polytechnic University, Erbil, 44001, Iraq; e Department of Mathematical and Statistical Methods, Poznań University of Life Sciences, Poznań, 60-637, Poland

**Keywords:** Genotype-by-environment interaction, Stability, AMMI model, Mahalanobis stability index

## Abstract

One of the most crucial challenges for finding superior crop genotypes with wide or specific adaptation throughout multi-environment trials (MET) is analyzing genotype-by-environment interaction (GEI) and phenotypic stability. In this study, an existing MET dataset––including 18 advanced barley genotypes along with two check cultivars evaluated across 10 environments (five locations in Iran over two consecutive cropping seasons)––previously analyzed with classical Additive Main Effects and Multiplicative Interaction (AMMI) and GGE biplot models was comprehensively reanalyzed using a Bayesian framework. Unlike classical frequentist approaches that rely on fixed point estimates without quantifying estimation uncertainty, the Bayesian paradigm incorporates prior distributions and posterior probability density intervals, effectively mitigating overfitting and providing robust probabilistic credibility for GEI parameters. The evaluation integrated Bayesian AMMI (B-AMMI), Bayesian Weighted Average of Absolute Scores (BWAAS), the combined performance-stability index (BWAASY), and Mahalanobis stability distance. According to the B-AMMI2 biplot, the first two interaction principal component axes (IPCA1 = 41.87% and IPCA2 = 24.35%) jointly accounted for 66.22% of the GEI variance. This revealed significant environmental discrimination (E1, E3, E4, E5, E9, and E10) and delineated a mega-environment (E7, E8, and E9). While genotypes G7, G9, G12, G15, and G16 revealed specific adaptations, genotypes G8, G11, G17, G18, and G19 displayed high stability near the origin. Posterior estimations of BWAAS confirmed that G2, G10, G11, G18, and G19 were the most stable. Beyond confirming previously recognized elite lines (G3, G10, and G14), the Bayesian framework offered superior discriminative resolution: simultaneous selection via BWAASY and Mahalanobis stability distance effectively resolved ambiguity in candidate selection, revealing previously obscured top-performing stable genotypes (e.g., G3, G10, and G14) and confirming G3, G4, and G10 as possessing optimal compromises between high yield and low GEI sensitivity, while precisely classifying G14 as specifically adapted rather than broadly stable. Overall, re-evaluating MET data under the Bayesian AMMI framework provided a powerful and statistically sound probabilistic basis for breeding programs to increase selection accuracy.

Specifications Table

**Table:** 

Subject	Biology
Specific subject area	Agronomy and Crop Science
Type of data	Table and Figure
Data collection	This dataset came from a multi-environment trial that used an RCBD with three replicates to assess 18 promising barley genotypes and two commercial cultivars during two cropping seasons (2021–2023) across five warm-climate stations. A Wintersteiger seed planter (Ried, Austria) was used to sow 350 seeds per square meter in six 5-meter rows spaced 20 cm apart. After removing border rows, plots were harvested using a Wintersteiger plot combine at physiological maturity to calculate grain yield (kg ha⁻¹). Using the ChiDO platform, Gibbs sampling (50,000 MCMC iterations, 5000 burn-in, thinning=5) was used to assess genotype-by-environment interaction (GEI), stability, and posterior parameters (BWAAS, BWAASY, MSD).
Data source location	Five localities in Iran's warm regions—Ahvaz (31°19′13′′ N 48°40′09′′ E), Darab (28°45′07′′ N 54°32′40′′ E), Zabol (37°15′00′′ N 55°10′02′′ E), Moghan (39°38′54′′ N 47°55′03′′ E), and Gonbad (31°01′43′′ N 61°30′04′′ E)—provided the experimental data.
Data accessibility	Repository name: B-AMMI dataset Data identification number: https://doi.org/10.17632/kf87fp2yrg.2 Direct URL to data: https://data.mendeley.com/drafts/kf87fp2yrg
Related research article	[1] S. Rahmati, R. Azizi-Nezhad, A. Pour-Aboughadareh, A. Etminan, L. Shooshtari. Analysis of genotypes-by-environment interaction effect in barley genotypes using AMMI and GGE biplot methods. Heliyon, 10:e38131. https://doi.org/10.1016/j.heliyon.2024.e38131

## 1 Value of the Data

• As a statistical benchmark for MET evaluation under Bayesian frameworks, this dataset offers thorough posterior estimates and greatest posterior density (HPD) intervals for yield and stability parameters (B-AMMI, BWAAS, BWAASY, and Mahalanobis stability distance).
• Mega-environment delineation and testing-network optimization are made easier by vector analysis and interaction patterns among five geographically different warm-climate stations throughout two consecutive seasons (2021–2023).
• This dataset directly supports parental selection and variety release in warm-region barley breeding programs by identifying new promising breeding genotypes (such as G3 and G4) in comparison to commercial checks (Oxin and Golchin).

## 2 Background

One of the main obstacles to crop genotype selection in any breeding program is the genotype × environment interaction (GEI) impact because genotype performance can differ greatly between environments. The genetic potential of a genotype (G), the environmental variables (E), and the interaction between G and E (GEI) influence phenotypic performance in multi-environment trials (METs). According to a major GEI, a genotype's response to environmental factors varies, potentially leading to variations in its relative ranking across environments (locations or years). Therefore, one of the primary goals of plant breeding projects is the identification of stable and high-yielding genotypes [2,3]. Barley (*Hordeum vulgare* L.), which is grown in marginal croplands under a variety of agroecological settings and is regularly subjected to significant variation in environmental parameters, such as temperature, precipitation, and soil characteristics, makes this issue more pertinent. It is crucial to take GEI into consideration when choosing superior genotypes because recent MET experiments in barley have demonstrated significant G, E, and GEI impacts for grain yield and other agronomic parameters [4,5].

To examine the GEI and assess genotype stability, a number of statistical techniques and models have been proposed, including the Finlay–Wilkinson method, the Eberhart–Russell approach, a set of parametric and non-parametric statistics, the additive main effects and multiplicative interaction (AMMI) model, and the genotype plus GEI (GGE) biplot approach [2,[6], [7], [8], [9]]. Among these, AMMI and GGE biplot techniques offer a helpful graphical depiction of GEI responses and enable the analysis of the primary and interaction effects within a single framework [10]. The use of AMMI and GGE biplot methods for detecting GEI and selecting genotypes that combine high grain production with desirable stability across diverse environments has been validated by recent studies on barley [[11], [12], [13], [14]]. The best linear unbiased prediction (BLUP) and linear mixed models (LMM), in addition to AMMI and GGE, have grown in significance in modern breeding programs. These models are especially useful when MET datasets have missing observations or are unbalanced because they enable the prediction of genotypic values while considering both fixed and random sources of variance. Additionally, these models offer genotypic performance estimates that are adjusted for shrinkage, which can increase the accuracy of selection choices. The combination of AMMI, BLUP, and GGE techniques has been demonstrated in a MET study to offer information for finding stable and high-yielding barley genotypes [15].

Although frequentist techniques have significant benefits, they typically rely on point estimates of genotype performance and stability. Because genotype performance is fundamentally unclear due to environmental variability, experimental error, missing data, and changing experimental accuracy across settings, this constraint presents a considerable problem in practical breeding efforts. Bayesian statistical techniques offer a strong framework for addressing this and quantifying the uncertainty related to genotype and GEI estimates. They do this by combining observed data with prior knowledge to produce posterior probability distributions for unknown parameters [16]. By incorporating Bayesian inference into the estimation of multiplicative interaction components and additive main effects, Bayesian AMMI (B-AMMI) expands upon the traditional AMMI model. Because the predicted interaction scores in the standard AMMI model are unclear, it is frequently difficult to draw conclusions about bilinear interaction parameters. A Bayesian approach to AMMI was proposed by Crossa et al. [16], in which Markov chain Monte Carlo (MCMC) techniques are used to derive the posterior distributions of model parameters. By incorporating credible or greatest posterior density (HPD) regions into AMMI biplots, this method enables a direct evaluation of GEI parameter uncertainty. As a result, B-AMMI offers a strong probabilistic framework for statistical inference on interaction components while maintaining the helpful decomposition of AMMI [16]. Therefore, B-AMMI should be seen as an extension that offers more statistical information on uncertainty and genotype–environment relationships rather than as a replacement for traditional AMMI, GGE, or BLUP approaches in crop breeding. Combining these methods can improve the accuracy of genotype selection and offer a more thorough understanding of the GEI. Specifically, the B-AMMI has the potential to change stability analysis from a straightforward ranking pattern into a probabilistic framework for decision-making where breeders can assess a genotype's expected performance as well as the probability and uncertainty surrounding its superiority and stability [15,16]. Therefore, this dataset shows how the B-AMMI model may better describe the stability of barley genotypes, especially by elucidating the actual performance and stability potential of the genotypes found in our earlier study [1].

## 3 Data Description

Four figures make up the dataset used in this research. The B-AMMI 2 biplot in Fig. 1 efficiently illustrates the GEI's complexity throughout the investigated contexts. Together, the first two interaction principal component axes (IPCA1 and IPCA2) explained 66.22% of the interaction pattern and explained 41.87% and 24.35% of the overall GEI variance, respectively. Genotypes G8, G11, G17, G18, and G19 showed the smallest distance from the biplot origin in terms of stability, showing strong relative stability throughout the assessed environments. The genotypes with the highest IPCA scores, G7, G9, G12, and G16, on the other hand, demonstrated adaptability rather than general stability. Environmental discrimination varied greatly; the GEI structure was most affected by E1, E3, E4, E5, E9, and E10 with long vectors. While E2, E3, and E10 showed more different environmental response patterns, angle analysis of environmental vectors showed high correlations among E7, E8, and E9, indicating a possible mega-environment. Certain genotype-environment relationships were evident: G16 showed possible adaptation to E10, G15 to E1, G9 to E3, and G7 to the lower-left environmental cluster (more precisely, E4 and E5).

**Fig. 1:**
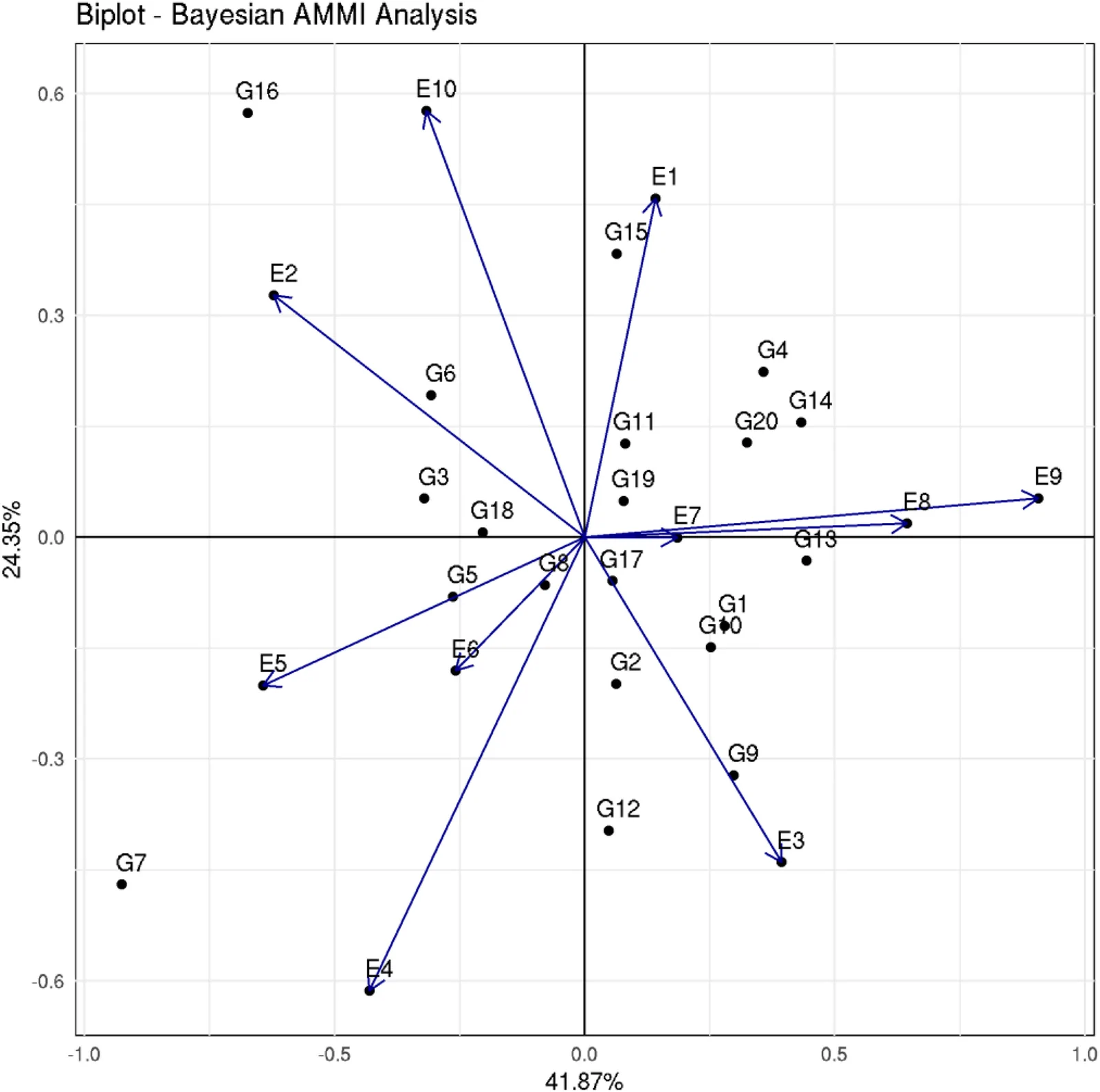
Bayesian-AMMI II biplot to show the impact of genotypes and environments on the GEI effect. E1/E2, E3/E4, E5/E6, E7/E8, and E9/E10 represented the first- and second-years experiments in the Ahvaz, Darab, Gonbad, Zabol, and Moghan locations, respectively.

The posterior estimates of the weighted average of absolute scores (WAAS) for each genotype, along with their uncertainty intervals, are displayed in Fig. 2's Bayesian WAAS (BWAAS) plot. Because it gauges the degree of GEI, this index serves as a stability indicator: (i) higher BWAAS values suggest more environmental sensitivity and greater specialized adaptation, and (ii) lower BWAAS values imply greater stability and more consistent performance across settings. As a result, the most stable genotypes were G2, G10, G11, G18, and G19, which had the lowest BWAAS values. Additionally, genotypes G3, G4, G8, G13, G17, and G20 displayed low-to-moderate values and might be regarded as fairly stable. G6, G7, G15, and G16, on the other hand, showed the highest BWAAS values. Because of their higher GEI, these genotypes were less stable in all the environments that were investigated. However, if they perform well in particular target situations, their high BWAAS values might also suggest a potentially helpful specific adaptation.

**Fig. 2:**
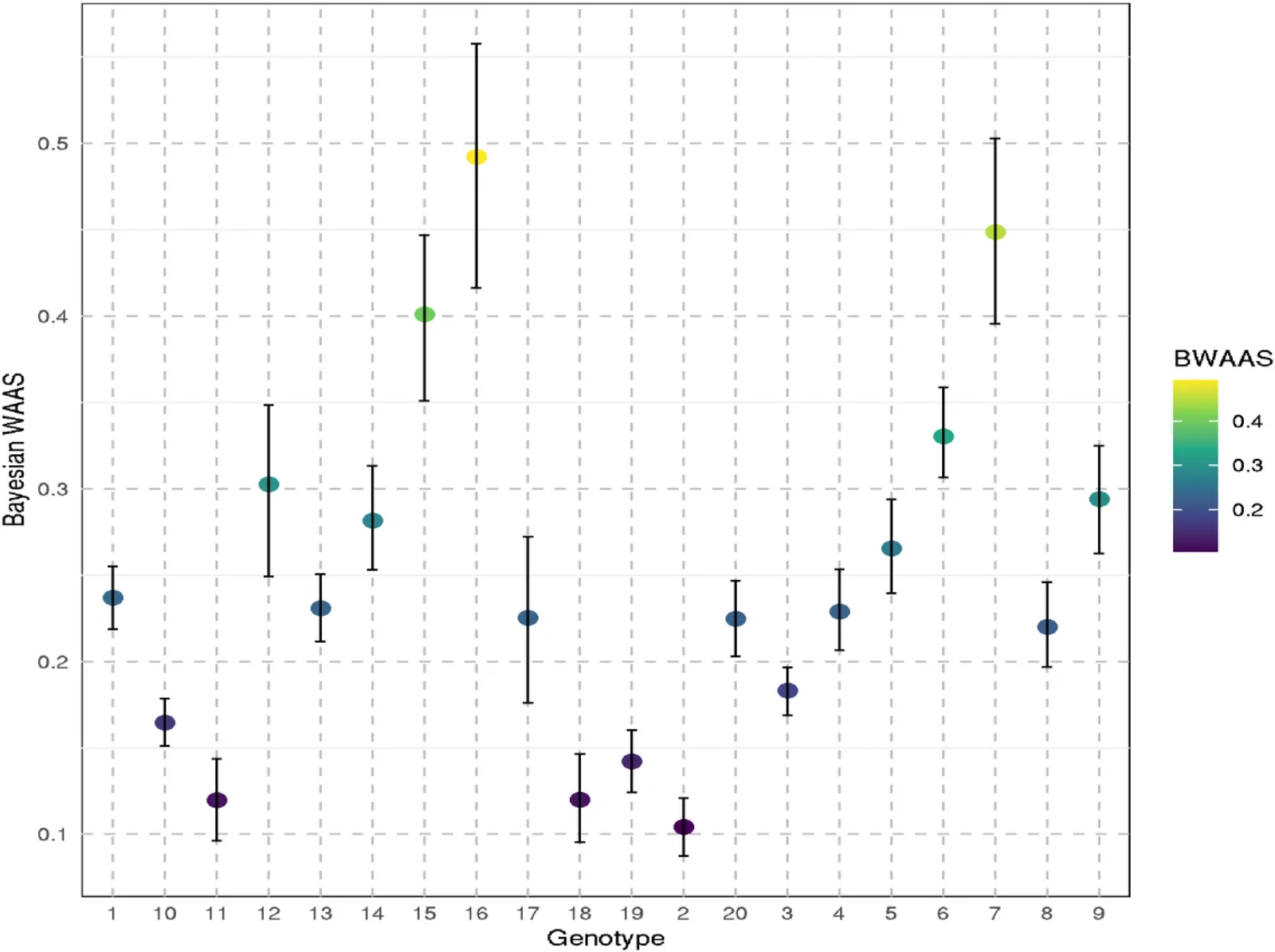
The genotypes' estimated Bayesian weighted average of absolute score (BWAAS) values.

A combined assessment of genotype performance and stability is given by the Bayesian WAASY plot (Fig. 3). Higher values in this kind of index typically indicate a more favorable mix of low GEI and high mean performance. Genotypes G3, G2, G10, G20, G11, G14, and G17 displayed the highest BWAASY values according to this index. G3 stands out among these since it has the lowest BWAAS value and the highest BWAASY value, indicating a favorable balance between stability and production. While G3 and G14 had high BWAASY values but considerably more interaction than G2 and G10, G10 also did well in the combined index and had comparatively low BWAAS. For G1, G4, G5, G8, G9, G13, G18, G19, and G20, intermediate BWAASY values were noted. Although the final ranking of these genotypes should be based on the economic significance of yield vs stability and the posterior uncertainty associated with the index, they may have a moderate overall appeal. G6, G7, G12, G15, and G16 had the lowest BWAASY scores. A combined yield-stability requirement makes these genotypes less appealing. Their somewhat high BWAAS estimates, especially for G7, G15, and G16, are in line with their low BWAASY values. Therefore, even if these genotypes might be specifically adapted to some situations, they don't seem to offer the best balance of performance and stability in all the environments.

**Fig. 3:**
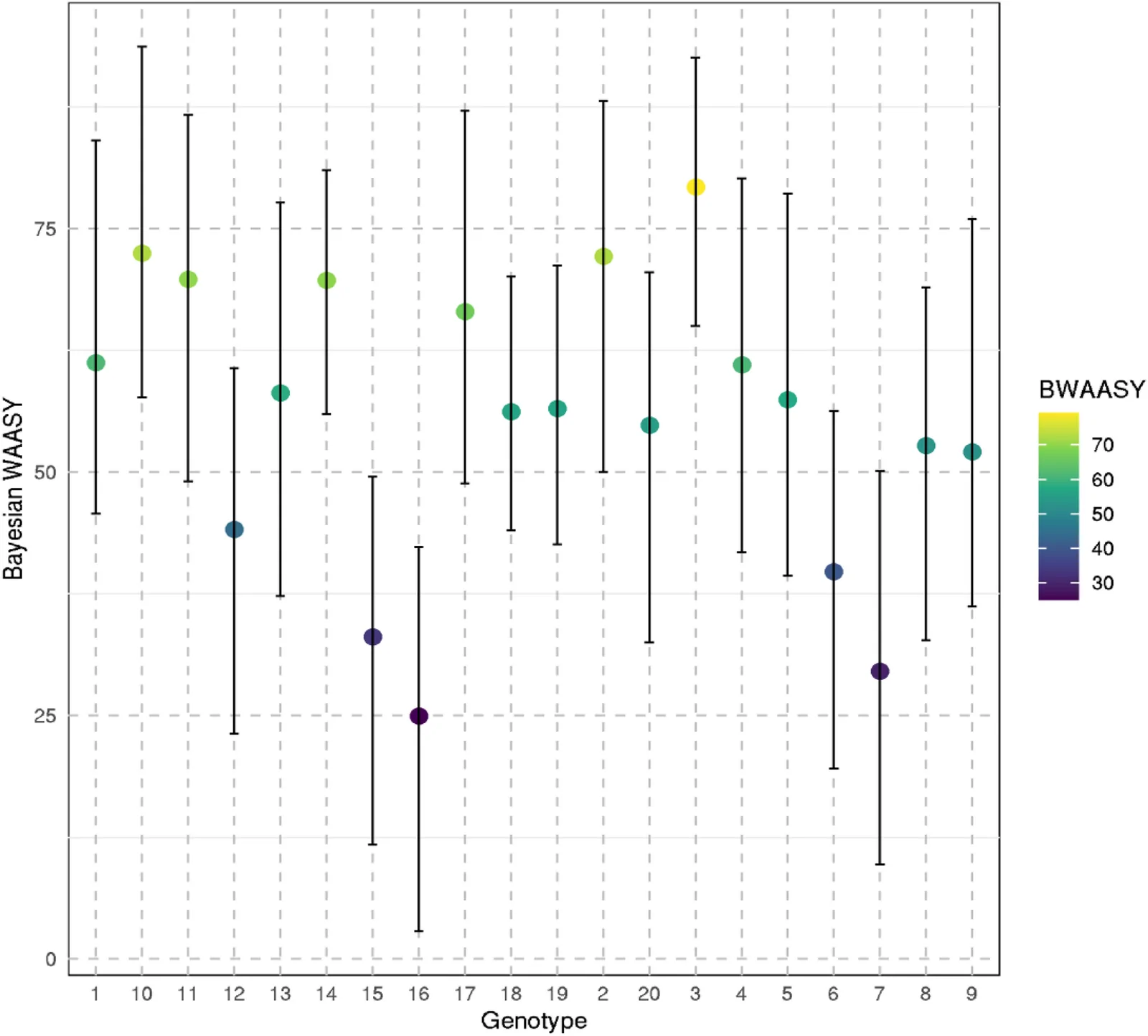
The genotypes' estimated Bayesian weighted average of absolute stability and mean performance (BWAASY) values were calculated using weights of 50 and 50 for yield and stability.

Significant genetic variation in stability and trait performance was shown by the Mahalanobis stability distance plot (Fig. 4). Genotypes G1, G3, and G4 were in the biplot's high-performance, low-distance area, suggesting a favorable combination of above-average posterior grain yield means and comparatively high stability. G3, which combined one of the higher posterior means with a low Mahalanobis stability distance, had the most appealing overall profile. G10 may be regarded as a stable genotype with somewhat favorable performance since it also showed a low stability distance. On the other hand, G9, G14, and G17 displayed higher Mahalanobis distances but comparatively high posterior means, indicating better performance together with increased environmental sensitivity and potential specialized adaptation. The genotypes G2, G11, G18, and G19 showed excellent stability but poor overall performance, with the lowest stability distances and below-average posterior means. G6, G7, G12, G15, and G16 were the least favorable profiles, typically combining high Mahalanobis distances with below-average or just moderate grain yield means. While G14 might be taken into consideration for specific adaptation, G1, G3, and G4 were shown to be the most promising choices overall for broad adaptation.

**Fig. 4:**
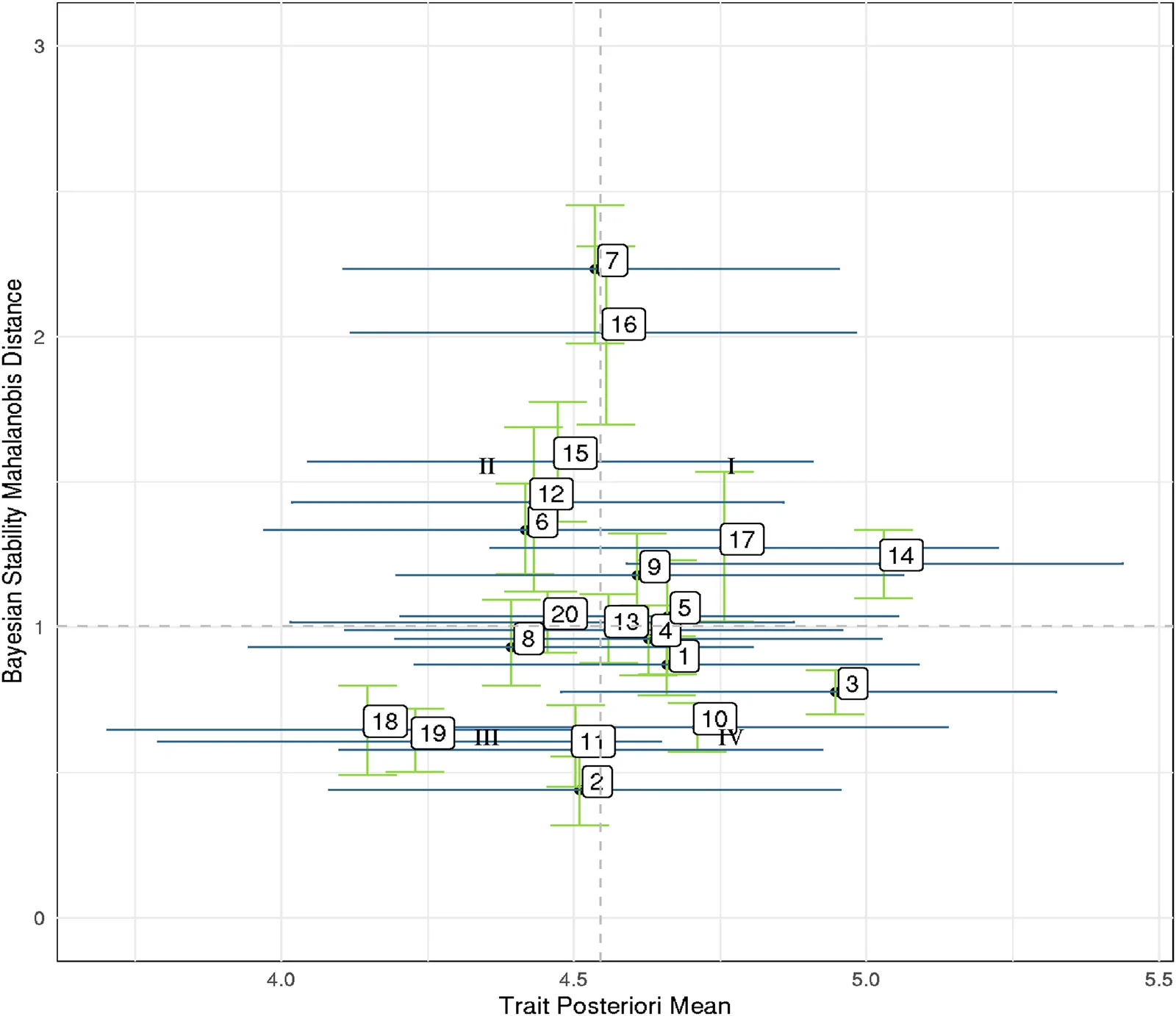
Grouping the evaluated barley genotypes based on the Bayesian Mahalanobis stability distance (MSD) index.

## 4 Experimental Design, Materials and Methods

The examination of two commercial cultivars (Oxin and Golchin) and eighteen advanced barley genotypes provided the data analyzed in this report. Iran's national irrigated barley breeding program for warm climates used field trials and domestic crossing procedures to select these genotypes. During the agricultural seasons of 2021–2023, METs were carried out at five research sites situated in Ahvaz, Darab, Zabol, Gonbad, and Moghan. Our earlier study [1] includes the pedigrees of the assessed genotypes as well as additional geographic data for the test conditions. The experiment was set up in a randomized complete block design (RCBD) with three replications for each environment. The plant materials were sown on fields that had been left fallow the year before in all environments. At each station, pre-sowing land preparation—including plowing, disking, and leveling—was carried out in accordance with professional recommendations. Urea, potassium, and triple superphosphate fertilizers were applied at rates of 32, 50, and 100 kg ha⁻¹, respectively, during soil preparation and before sowing. Each experimental plot in every trial was made up of six 5-meter rows spaced 20 cm apart. Sowing was done in November using a plot seed planter (Wintersteiger, Ried, Austria) at a seeding density of 350 seeds m⁻², depending on the site's environmental circumstances. Puma Super and Granstar herbicides were used to manage weed growth during the stages of plant growth and development. Additionally, at the stem elongation stage, an extra 40 kg ha⁻¹ of urea was sprayed. Throughout the growing season, every crop-management procedure was carried out in compliance with conventional expert guidelines. After eliminating border effects, all experimental plots were harvested using a plot combine (Wintersteiger, Ried, Austria) at the physiological maturity time. Grain yield was measured in tonnes per hectare (ton ha⁻¹) using the harvested grains for each genotype in each environment.

The Bayesian AMMI (B-AMMI) model was used to characterize the GEI structure. Using procedures outlined by Olivoto et al. [17], the weighted average of absolute scores (WAAS) and its yield-weighted equivalent (WAASY) were calculated based on the B-AMMI model:

$$\text{WAAS}=\;\sum_{k=1}^{p}\left|IPCA_{ik}\;\cdot EP_{k}\right|\;/\;\sum_{k=1}^{p}EP_{k}$$

$$\text{WAASY}\;=\;\frac{\left(rG_{i}\;\cdot \theta_{Y}\right)\;+\;\left(rW_{i}\;\cdot \theta_{S}\right)}{\theta_{Y}\;+\;\theta_{S}}$$

where $rG_{i}$ is the rescaled values (0–100) for grain yield, $rW_{i}$ is the rescaled values (0–100) for WAASY, $\theta_{Y}$ is the weight for grain yield, and $\theta_{S}$ is the weight for WAASY index. IPCAik is the score of the *i* th genotype in the *k*-th IPCA, and $EP_{k}$ is the explained variance of the *k*-th IPCA for 1, 2,…, and *p*. According to Johnson and Wicherm [18], the Mahalanobis stability distance (MSD) measure was used to further assess genotypic stability in connection to an ideotype. In this approach, the ideotype is similar to a hypothetical genotype located at the origin of the interaction main component space. The following formula was used to determine the MSD index:

$$\text{MSD}\;=\;{\left(IPCA_{i}-\;0\right)}^{T}\Sigma^{-1}\left(IPCA_{i}-0\right)$$

where Σ is a diagonal matrix made up of the reciprocals of eigen-values from the interaction term of the AMMI model, $IPCA_{i}$ is the *i* th *p*-dimensional vector of scores from the interaction term of the AMMI model, and 0 is the p-dimensional vector of zero, which represents the ideal genotype regarding the stability (origin). Lower MSD index values are indicative of greater genotypic stability. The Mahalanobis distance considers the importance of IPCAs through the diagonal matrix Σ. This matrix contains the reciprocals of the eigenvalues associated with each IPCA. The eigenvalues itself indicate the extent to which the related IPCA can account for the variance from the AMMI model's interaction term. The Mahalanobis stability plot shows the expected MSD index values for each genotype along with the corresponding marginal posterior means for yield (or other attribute of interest). Additionally, it provided a comprehensive grasp of the estimates and uncertainty of the Bayesian model by displaying the 95% highest posterior density (HPD) intervals for yield and MSD. The B-AMMI model was fitted for Bayesian inference using the Gibbs sampler algorithm with 50,000 Markov chain Monte Carlo (MCMC) iterations, which included a burn-in of 5000 iterations and thinning every five steps. The posterior distributions of AMMI-based indexes, such as the MSD index, Bayesian weighted average of absolute scores (BWAAS), and Bayesian weighted average of absolute scores integrating yield (BWAASY), were directly computed using the sampling parameters **λ** and **α**. All analyzes were carried out using the ChiDO tool (https://jarquinlab.shinyapps.io/chido/) [19].

## Limitations

Not applicable

## Ethics Statement

All authors have read and follow the ethical requirements for publication in Data in Brief and our work meets these requirements. Our work does not involve studies with animals and humans.

## CRediT authorship contribution statement

**Salim Rahmati:** Methodology, Investigation. **Alireza Pour-Aboughadareh:** Conceptualization, Resources, Software, Validation, Writing – review & editing. **Reza Azizi-Nezhad:** Data curation, Writing – review & editing. **Lia Shooshtari:** Conceptualization, Methodology. **Alireza Etminan:** Validation, Conceptualization. **Bita Jamshidi:** Software, Writing – original draft. **Omid Jadidi:** Software. **Jan Bocianowski:** Validation, Writing – review & editing.
